# Supplementary material for: The RNA-binding protein HuR is a negative regulator in adipogenesis
Source: Nat Commun. 2020 Jan 10;11:213. doi: 10.1038/s41467-019-14001-8 (PMC6954112; doi:10.1038/s41467-019-14001-8)
Supplement: Supplementary file 1 — Supplementary Information [file 41467_2019_14001_MOESM1_ESM.pdf]

Supplementary Information

The RNA-binding protein HuR is a negative regulator in adipogenesis

Siang et al.

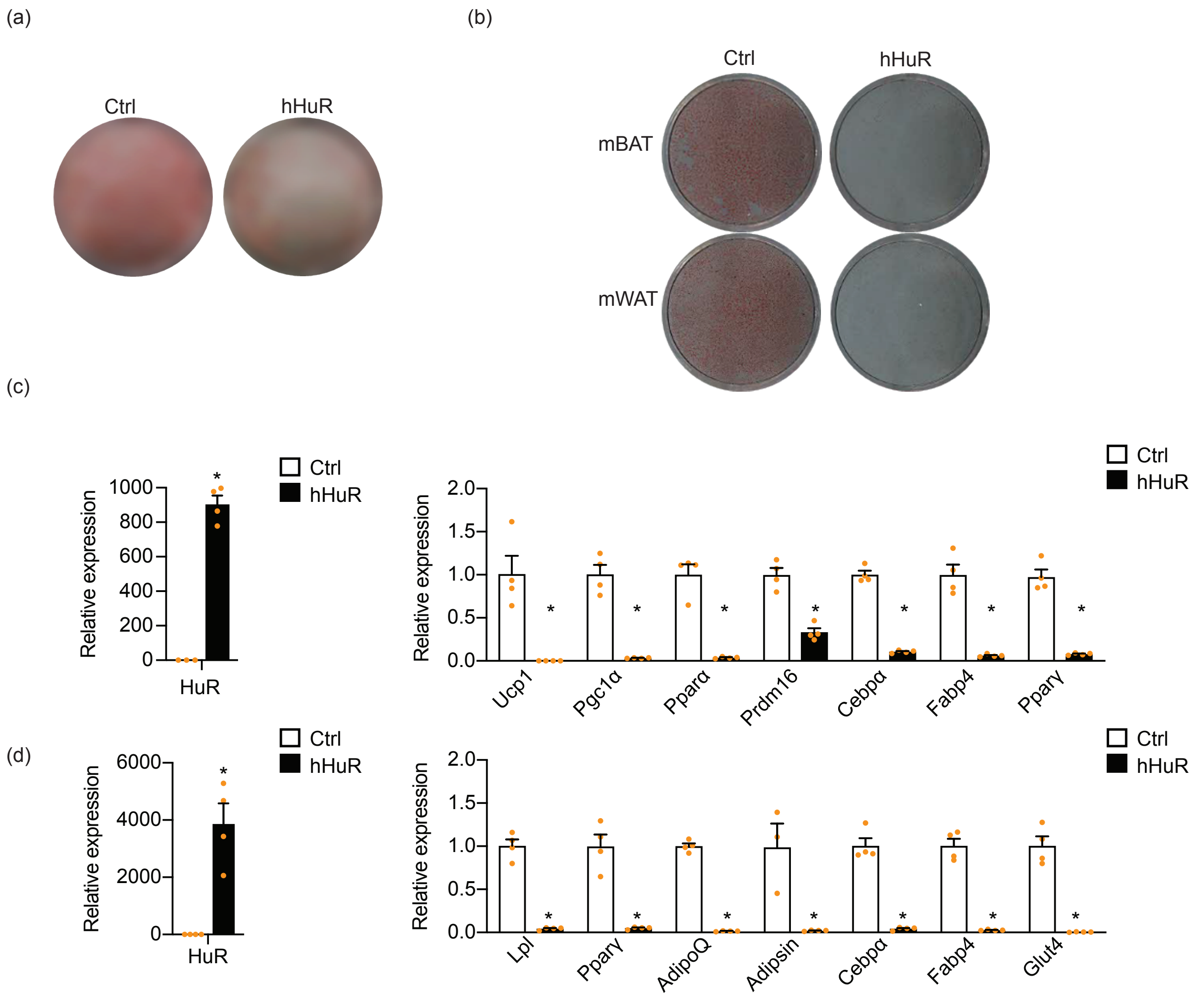

Supplementary Fig.1 Human HuR overexpression inhibits the differentiation of mouse adipocytes. (a) Retroviral overexpression of Human HuR in primary white preadipocytes, followed by induction of differentiation for 14 days. Oil red O staining was conducted to stain the lipid contents. (b) Oil red O staining for mouse brown adipocytes (Upper) or white adipocytes (Lower) overexpressing human HuR. (c) Real-time PCR to examine HuR (left) and marker gene expression (right) in brown adipocyte culture expressing human HuR after 5 days of differentiation. n=4 per group. (d) Similar as in (c), but in primary white adipocyte culture. n=4 per group, Error bars are mean ± SEM, Statistical significance was determined by Student's *t*-test; *p<0.05.

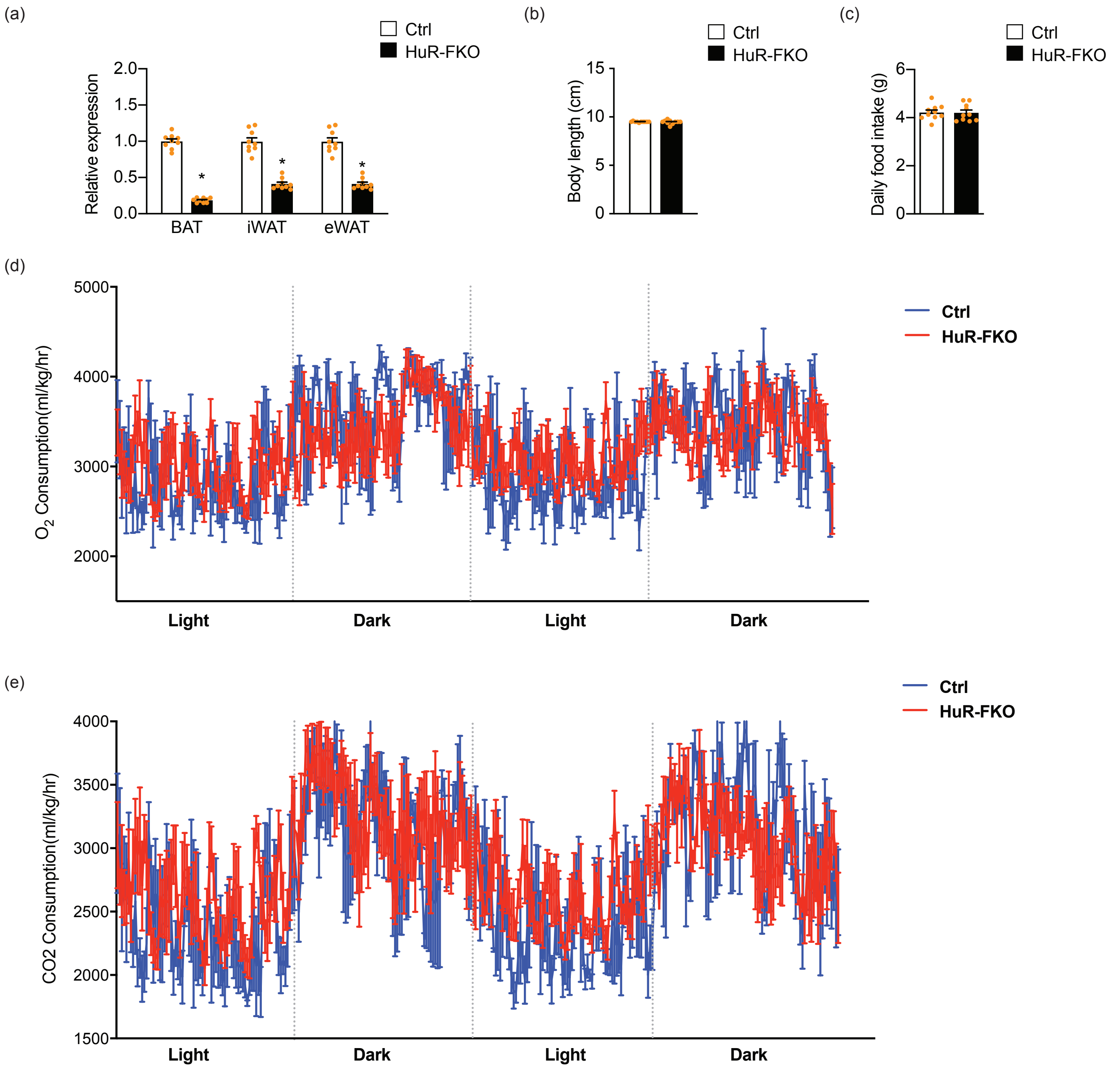

Supplementary Fig.2 Adipose-selective deletion of HuR doesn't affect animals' body weight, food intake and oxygen consumption. (a) Real-time PCR to examine HuR expression in BAT, iWAT and eWAT of HuR-FKO and control mice. n=9 per group. (b) Body length of 3-month-old male HuR-FKO and control mice. n=10 per group. (c) Daily food intake of 3-month-old male HuR-KFO and control mice. n=10 per group. (d-e) Oxygen consumption in the control and HuR-KFO mice measured in CLAMS cages over 48 hours period (Ctrl: n=3; HuR-BATKO: n=5), Error bars are mean ± SEM, Statistical significance was determined by Student's *t*-test; *p<0.05.

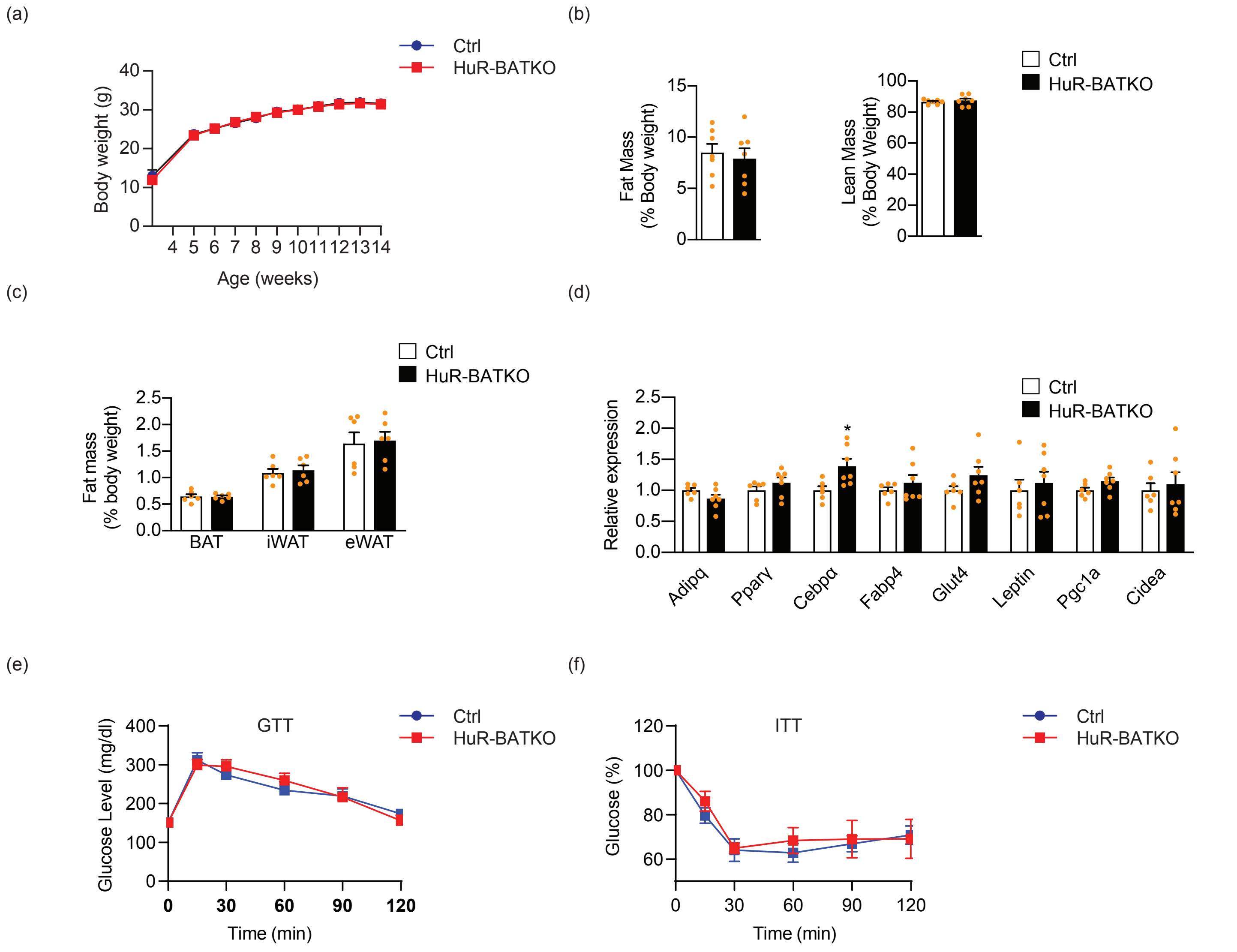

Supplementary Fig.3 Deletion of HuR in BAT doesn't affect animals' body weight and fat mass. (a) Growth curves of HuR-BATKO and Control male mice on chow diet. (Ctrl: n=8; HuR-BATKO: n=7). (b) In vivo body composition by EcoMRI of fat mass (left) and lean mass (right) in 4-month-old male HuR-BATKO and control littermates. n=7 per group (c) The organ weights of BAT, iWAT and eWAT in 4-month-old HuR-BATKO and Control littermates were normalized as a percentage of total body weight (n=6 per group). (d) Real-time PCR analysis of marker gene expression in eWAT (Ctrl: n=6; HuR-BATKO: n=7). (e) Blood glucose levels during glucose tolerance test (GTT) and (f) Insulin tolerance test (ITT) of HuR-BATKO and control littermates (Ctrl: n=8; HuR-BATKO: n=7). Error bars are mean ± SEM, Statistical significance was determined by Student's *t*-test; *p<0.05.

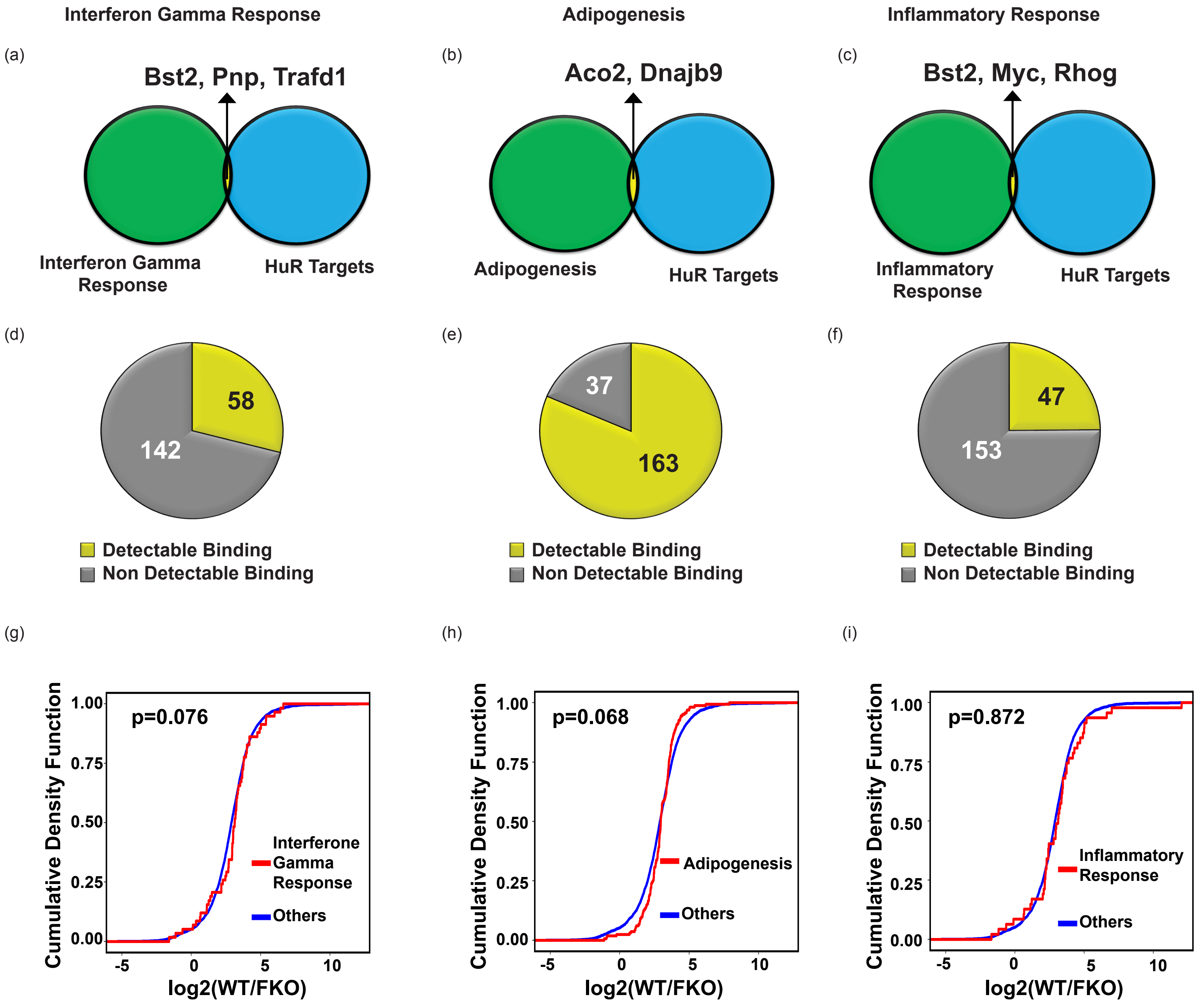

Supplementary Fig.4 Overlaps of the HuR targets with the genes in the GSEA.
(a-c) Overlaps of HuR targets with downloaded MSignDB reference gene sets of (a) Hallmark_Interferon Gamma Response, (b) Hallmark_Adipogenesis and (c) Hallmark_Inflamatory Response. (c-d) Proportion of genes within reference gene sets of (d) Hallmark_Interferon Gamma Response, (e) Hallmark_Adipogenesis and (f) Hallmark_Inflamatory Response with detectable HuR binding. (g-i) Cumulative density functions of the genes with detectable HuR binding identified from (d-f) for (g) Hallmark_Interferon Gamma Response, (h) Hallmark_Adipogenesis and (i) Hallmark_Inflamatory Response.

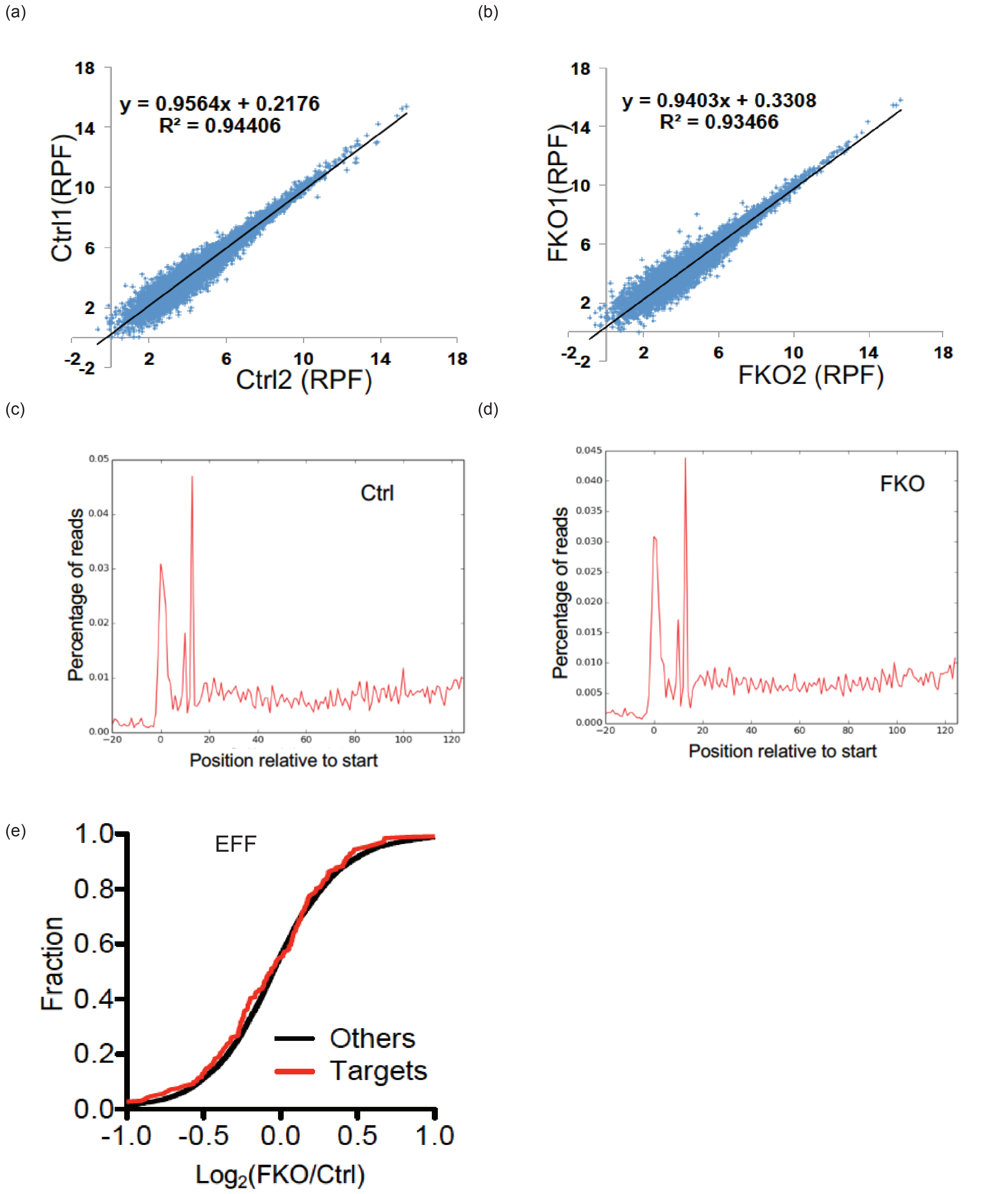

Supplementary Fig.5 Ribosome profiling analysis of the effect of HuR knockout in eWAT.
(a, b) Correlation of the ribosome protected fragments (RFP) between the duplicate samples in (a) Ctrl and (b) HuR-FKO eWATs. (c-d) Metagene analysis of translation initiation and termination. Average ribosome footprint density profiles of all the detectable mRNAs in eWAT from 6-weeks old (c) Ctrl and (d) FKO mice are aligned at their start codon and stop codon. (e) The cumulative fraction of the translational efficiency (RFP/FPKM) fold changes between FKO and Ctrl eWAT samples for HuR targets and other genes.

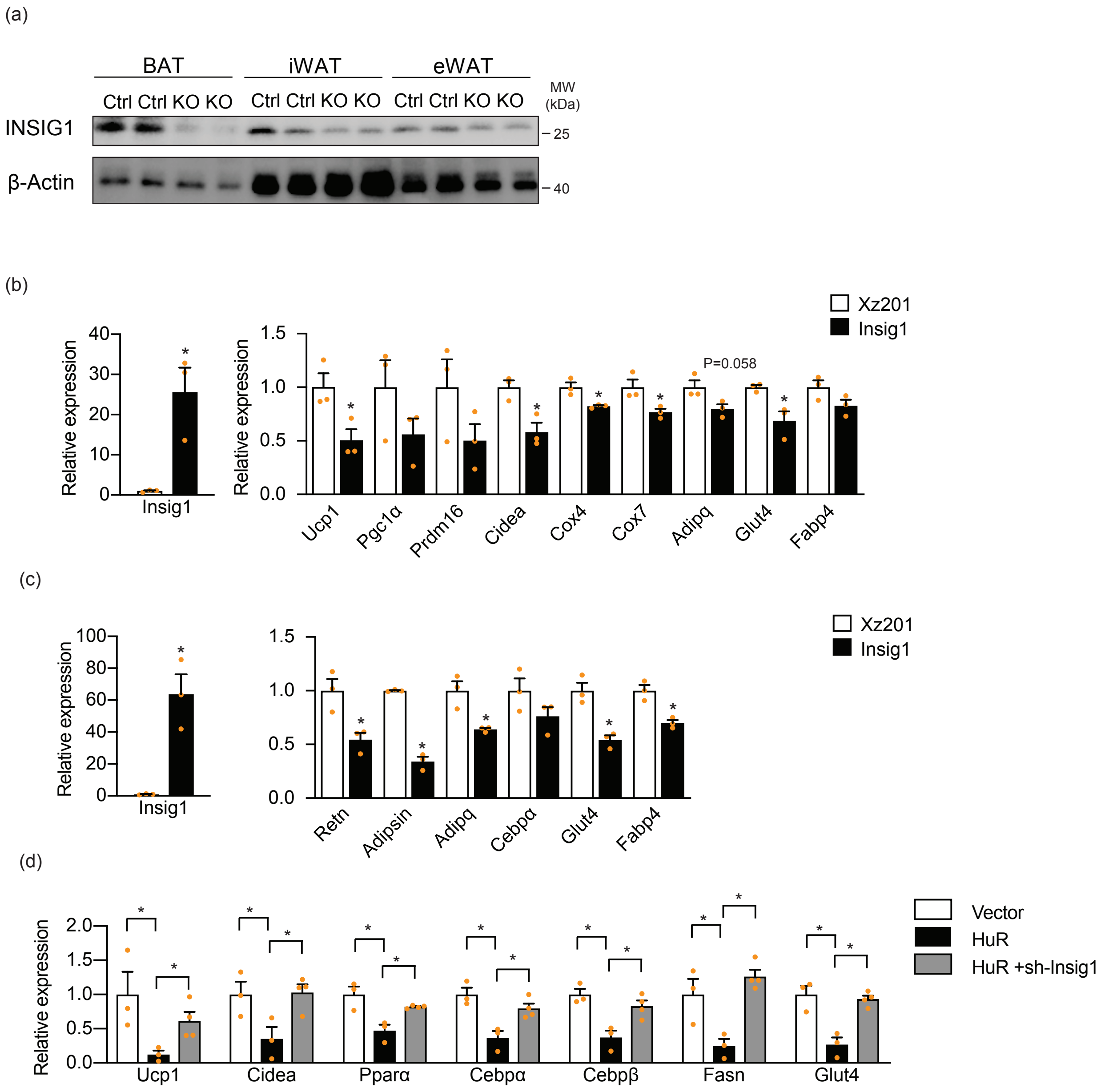

Supplementary Fig.6 Insig1 is a negative regulator in adipogenesis.
(a) Western blot analysis of insig1 expression in different adipose depots of Ctrl and HuR-FKO mice. (b) Retroviral overexpression of Xz201 (control vector) or insig1 in primary brown preadipocytes, followed by induction of differentiation for 5 days. Real-time PCR was conducted to examine Insig1 (left) and marker gene expression (right). n=3 per group. (c) Similar as in (b), but in primary white adipocyte culture. n=3 per group. (d) Retroviral overexpression of HuR and knockdown of insig1 in primary white preadipocytes, followed by induction of differentiation. Marker genes were examined by real-time PCR at day 5 (Vector: n=3; HuR overexpression: n=3; HuR+sh-Insig1: n=4). The error bars are mean ±SEM, Statistical significance was determined by Student's *t*-test; *P<0.05.

(a)

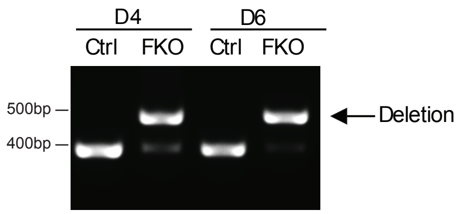

(b)

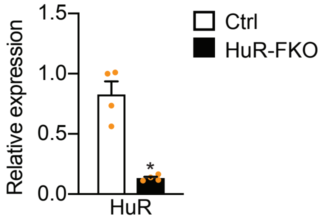

Supplementary Fig.7 Deletion efficiency of HuR in FKO-HuR adipocyte culture *in vitro.*
(a) Subcutaneous WAT preadipocytes were isolated from FKO-HuR and control mice for *in vitro* differentiation. PCR analysis of genomic DNA from white adipocytes at Day 4 and Day 6 indicated HuR deletion efficiency. (b) Real-time PCR to examine the expression level of HuR in the control and HuR knockout adipocyte culture. n=4 per group. Error bars are mean ± SEM, Statistical significance was determined by Student's *t*-test; *p<0.05.

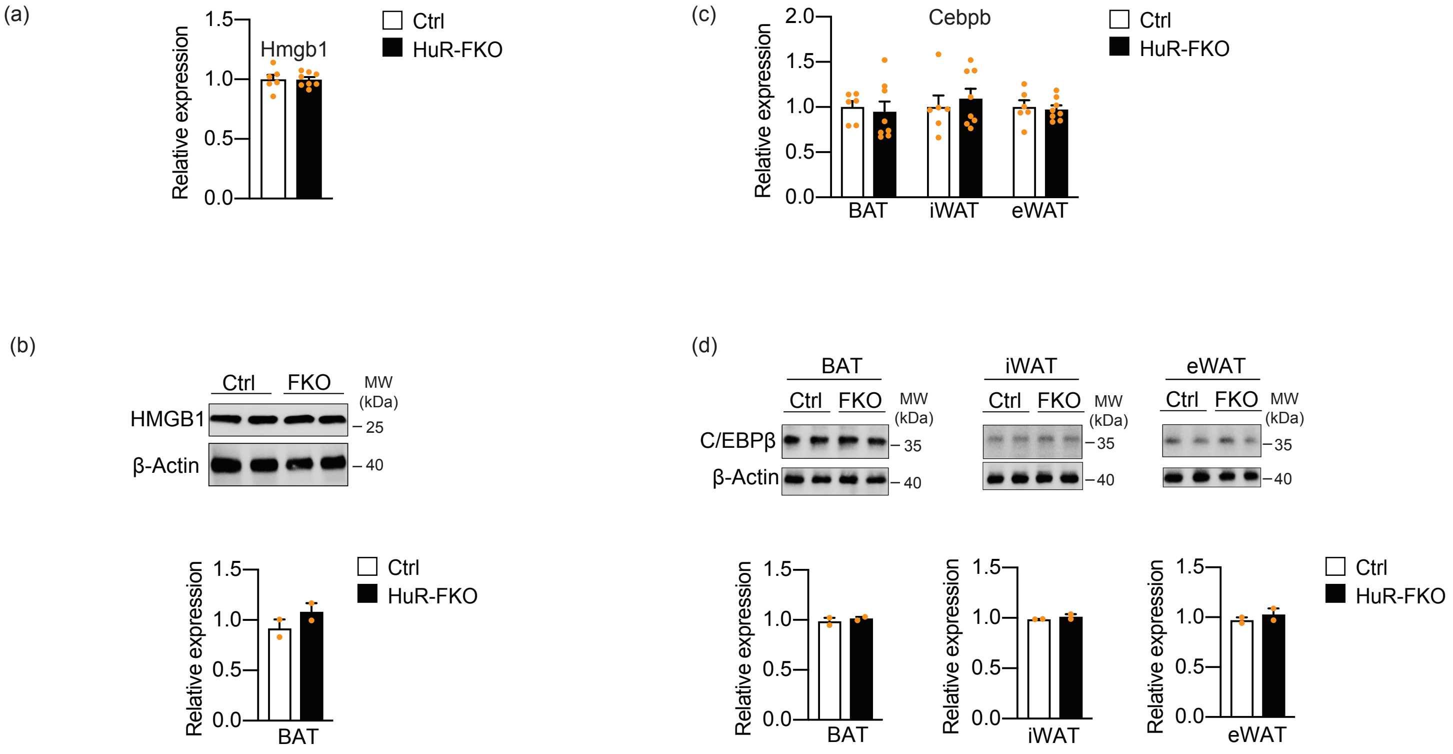

Supplementary Fig.8 The expression of two previously reported targets in HuR-FKO mice.
(a) The expression of Hmgb1 in BAT of HuR-FKO and control mice was examined by Real-time PCR (Ctrl: n=6; HuR-BATKO: n=8). (b) The western blot analysis (top) and quantification of the western blot for HMGB1 in BAT of control and knockout animals. The expression of HMGB1 is normalized to β-actin expression and their relative expression is shown (bottom) (n=2). The experiments were repeated twice using different animals. (c) The expression of Cebpb in BAT, iWAT and eWAT of HuR-FKO and control mice was examined by Real-time PCR (Ctrl: n=6; HuR-BATKO: n=8) and (d) Western blot (top). The expression of C/EBPβ is normalized to β-actin expression and their relative expression is shown (bottom) (n=2). The experiments were repeated at least twice using different animals. Error bars are mean ± SEM, Statistical significance was determined by Student's *t*-test.

(a)

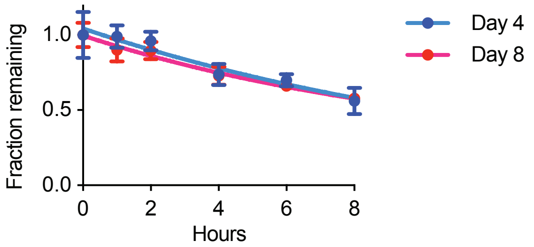

(b)

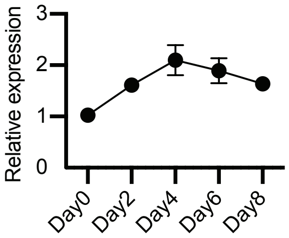

Supplementary Fig. 9 The regulation of HuR expression during adipogenesis.
Primary white preadipocytes were isolated from 3-week-old C57Bl6 mice for culture and then induced to differentiate for 8 days. (a) Actinomycin D was added to track the decay rate at day 4 and day 8. n=6. (b) Real-time PCR analysis of HuR expression at Day 0, 2, 4, 6 and 8. n=4 per group. Error bars are mean ± SEM.
