# Supplementary material for: The RNA-binding protein HuR is a negative regulator in adipogenesis
Source: Nat Commun. 2020 Jan 10;11:213. doi: 10.1038/s41467-019-14001-8 (PMC6954112; doi:10.1038/s41467-019-14001-8)
Supplement: Supplementary file 3 — Description of Additional Supplementary Files [file 41467_2019_14001_MOESM3_ESM.pdf]

Description of Additional Supplementary Files

File name: Supplementary Data 1
Description: qPCR Primer sequences.

File name: Supplementary Data 2
Description: Ribosome protected fragment from Control and FKO mice.

File name: Supplementary Data 3
Description: The gene expression of HuR targets

File name: Supplementary Data 4
Description: HuR immunoprecipitation followed by RNA sequencing.

File name: Supplementary Data 5
Description: AU-rich elements (ARE) presentation in HuR targets.

File name: Supplementary Data 6
Description: Oligos for cloning.
